# Structured patterns of activity in pulse-coupled oscillator networks with varied connectivity

**DOI:** 10.1371/journal.pone.0256034

**Published:** 2021-08-11

**Authors:** Kyra L. Kadhim, Ann M. Hermundstad, Kevin S. Brown

**Affiliations:** 1 Department of Chemical, Biological, and Environmental Engineering, Oregon State University, Corvallis, OR, United States of America; 2 Janelia Research Campus, Howard Hughes Medical Institute, Ashburn, VA, United States of America; 3 Department of Pharmaceutical Sciences, Oregon State University, Corvallis, OR, United States of America; Universidad Rey Juan Carlos, SPAIN

## Abstract

Identifying coordinated activity within complex systems is essential to linking their structure and function. We study collective activity in networks of pulse-coupled oscillators that have variable network connectivity and integrate-and-fire dynamics. Starting from random initial conditions, we see the emergence of three broad classes of behaviors that differ in their collective spiking statistics. In the first class (“temporally-irregular”), all nodes have variable inter-spike intervals, and the resulting firing patterns are irregular. In the second (“temporally-regular”), the network generates a coherent, repeating pattern of activity in which all nodes fire with the same constant inter-spike interval. In the third (“chimeric”), subgroups of coherently-firing nodes coexist with temporally-irregular nodes. Chimera states have previously been observed in networks of oscillators; here, we find that the notions of temporally-regular and chimeric states encompass a much richer set of dynamical patterns than has yet been described. We also find that degree heterogeneity and connection density have a strong effect on the resulting state: in binomial random networks, high degree variance and intermediate connection density tend to produce temporally-irregular dynamics, while low degree variance and high connection density tend to produce temporally-regular dynamics. Chimera states arise with more frequency in networks with intermediate degree variance and either high or low connection densities. Finally, we demonstrate that a normalized compression distance, computed via the Lempel-Ziv complexity of nodal spike trains, can be used to distinguish these three classes of behavior even when the phase relationship between nodes is arbitrary.

## Introduction

Many biological systems exhibit coordinated dynamics that are thought to underlie collective function. For example, organism-level physiological processes such as heart beats, neural activity, and circadian rhythms [1] along with population collective behaviours like quorum sensing [2] all exhibit patterns of coordinated activity, and the disruption of this coordination can be detrimental to system function [1, 3]. However, the precise nature of this coordination can take many forms, and there is no single convention for defining and characterizing the degree of coordination in complex dynamical systems.

This problem has been extensively studied in the context of coupled oscillator models, in which coordinated dynamics can emerge as a synchronous state of the system. In the Kuramoto model [4], a network of phase oscillators is said to be synchronized if all oscillators have identical phases. In networks of pulse-coupled oscillators, synchrony has been analogously defined as the state in which all oscillators fire in unison, and reliably emerges in networks with all-to-all connectivity, regardless of initial conditions [5]. So-called ‘chimera’ states, in which synchrony and asynchrony coexist, have also been observed in networks of identical phase oscillators with non-local connectivity [6–9], random connectivity [10], and modular connectivity [11], as well as in networks of neurons with other intrinsic dynamics [12–15].

Several studies have proposed methods of quantifying the degree of synchronous activity in limit cycle oscillator networks that rely on knowing the phases of all oscillators throughout time. These include various order parameters [4, 7, 16], measures of dispersion [9, 11], classical indicators of chaotic behavior [17–19], and local curvature [20]. However, these methods cannot be readily used to study spiking dynamics in pulse-coupled oscillator networks without knowledge of oscillator phases throughout time.

This problem becomes more difficult when studying pulse-coupled oscillator networks with varied connectivity, because the space of possible dynamical patterns is large. Several studies have explored how network dynamics depend on various topological properties, including connection density, network size, and degree heterogeneity. However, these studies have used different neural intrinsic dynamics (i.e. theta neurons [21] or leaky integrate-and-fire neurons [22]), different size ranges that do not overlap [23, 24], and different methods of quantifying synchronization [21–24]. It thus remains unclear how network connectivity shapes the space of dynamical activity patterns in networks of pulse-coupled oscillators.

Here, we study the emergence of structured dynamical patterns in networks of pulse-coupled oscillators with varied connectivity, and we show that these patterns fall into three broad classes that we call temporally-regular, temporally-irregular, or chimeric. We first show that these different classes can be characterized by the mean and variance of the inter-spike intervals (ISIs) of individual oscillators in the network. Using this method, we identify structural properties that influence the likelihood of finding network dynamics from each of these three classes. We then introduce a more multivariate measure that can be used to identify groups of coordinated oscillators and similarly classify the dynamics of the network. This new method is inherently more robust to variation in precise spike times, and thus could be more powerful than ISI statistics in the analysis of real neural spike trains.

## Methods

We study the dynamics of undirected, binomial random (i.e., Erdös-Rényi [25]) networks of pulse oscillators. We use the *G*(*N*, *p*) ensemble in which one specifies the number *N* of nodes in the network and the probability *p* that any two nodes are connected by an edge [26]. We constructed networks with fixed size *N* = 16 but varying edge probability *p*. For a given network, the value of *p* was drawn from a uniform distribution on the interval [0.2, 0.9] in cases where *p* is not specified. Any random network consisting of two or more disconnected components was excluded from analysis.

Each node in the network is a pulse oscillator with intrinsic dynamics of Mirollo-Strogatz [5] type:

$$\begin{array}{c} \dot{y}\left(t\right)=-y\left(t\right)+2. \end{array}$$ (1)

When an oscillator crosses unity, it “fires a spike” and resets to zero; we record these spike times and use them to assess patterns of network activity.

Oscillators are coupled through the exchange of pulses; each time an oscillator fires, it delivers a pulse of amplitude *ε* to each of its network neighbors. We fix *ε* = 1/(*N* − 1). Scaling *ε* with network size ensures that the largest pulse that an individual oscillator can receive in a given time-step is equal to unity; which would occur if that oscillator were fully connected. Reducing *ε* results in less firing activity, because nodes become dominated by their own intrinsic dynamics. In the limit as *ε* approaches zero, each node fires according to its own intrinsic dynamics and is not influenced by other nodes in the network. As we will later show, this particular scaling of *ε* compensates for an increase in mean degree as we scale the size of binomial random networks, and thus prevents a corresponding increase in the average pulse size to a given node were *ε* held constant.

**Fig 1:**
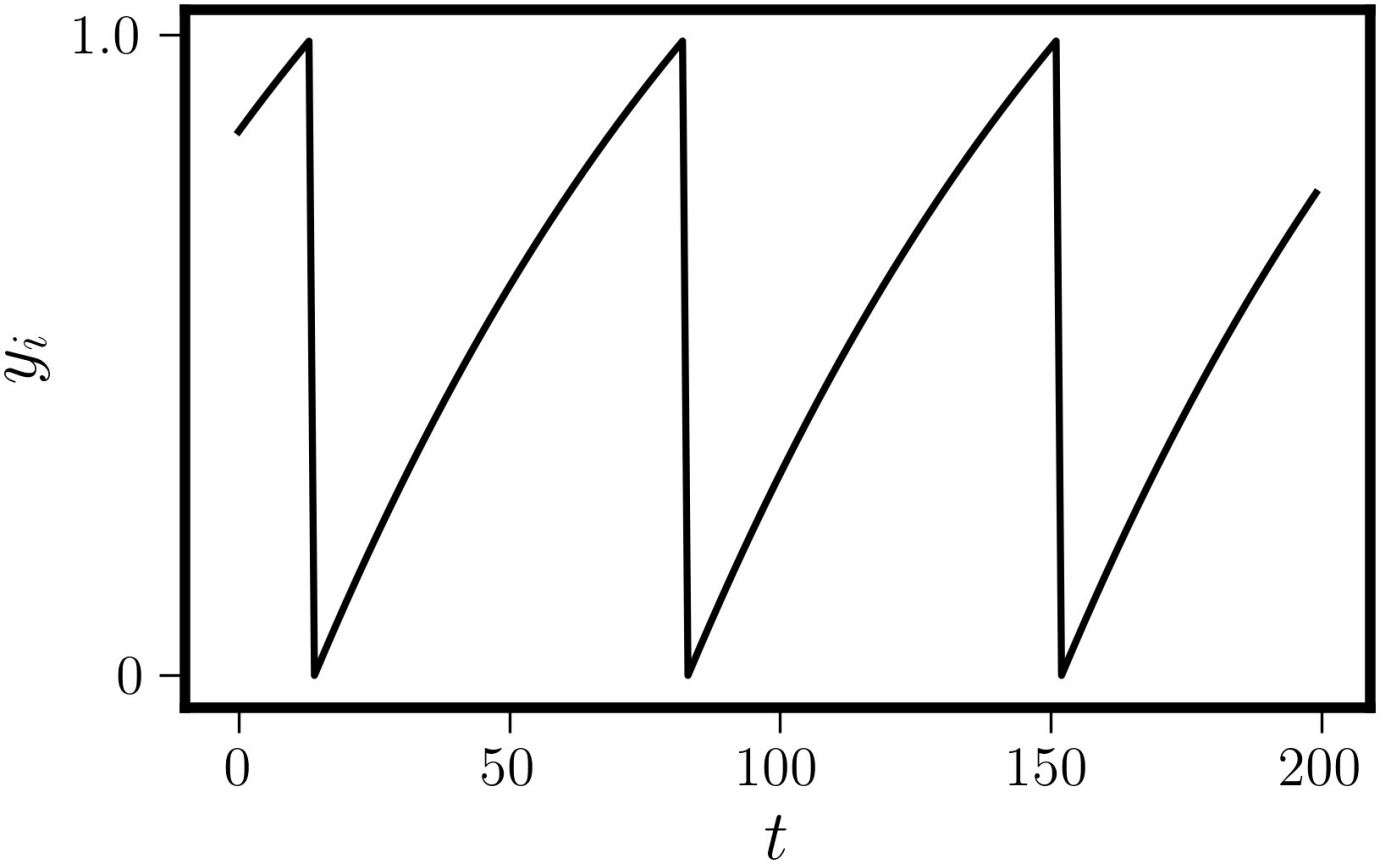
Nodal intrinsic dynamics. The first 200 time-steps of Eq 1. The node spikes when the activity crosses a threshold of 1, and then resets to a baseline of 0.

To simulate network dynamics, we use the Euler method [27] with a step size small enough that results were unaffected by cutting the step size in half and repeating the simulation. We initialize each network with random initial conditions drawn from a uniform distribution on the interval [0, 1] and then simulate the dynamics for 10,000 time-steps. We restrict our analysis to the final 2,000 time-steps of the simulation in order to ensure that the network dynamics have stabilized. There is only one real timescale in the problem: the intrinsic period of a node. For the parameter choices we make in Eq 1, the intrinsic period is 69 time-steps when integrated for a total time of 100 with 10,000 time-steps. All other times in the problem could be rescaled by the intrinsic period. The intrinsic dynamics of an individual oscillator are shown in Fig 1.

After an oscillator fires, we allow one time-step for the emitted pulse to reach its neighbors. For physical systems, this convention is more realistic than allowing immediate pulse transmission because it eliminates the possibility of chain-firing and accounts for the time required for signal transmission. In our simulations, it is possible to adjust the time of inter-node signal transmission to allow delays of any duration, something we do not explore here. Algorithm 1 specifies how we simulate the network dynamics.

**Algorithm 1**: Oscillator Dynamics Simulation

initialization

**for** *each time-step* **do**

 Note which oscillators are at or above threshold;

 **for** *each node* **do**

  Reset nodes above threshold to zero;

  Add spike to array for given node and time-step;

  Send pulses to nodes connected to those that just fired, added to the next time-step;

  Add integration step to the next time-step

 **end**

**end**

## Results

We propose two methods for identifying structured dynamical patterns in networks of pulse-coupled oscillators: inter-spike interval (ISI) statistics and normalized compression distances (NCDs). We explore how each of these methods can be used to characterize the dynamical state space of binomial random networks, and we then investigate their potential for characterizing the state space of other random graphs.

### Inter-spike interval statistics

Using spike times to calculate the distribution of ISIs for each node in the network, we observed that networks can be grouped into three broad classes that are defined by ISI mean and variance:

1. **Temporally-irregular**: all nodes have different ISI means and variances.
2. **Temporally-regular**: all nodes have the same ISI mean and variance.
3. **Chimeric**: at least one subset of nodes has a common ISI mean and variance, while the remaining nodes have different ISI means and variances.

To specify that two or more nodes have the same ISI statistics, we require that their ISI mean and variance be identical out to eight decimal places. We use both mean and variance to characterize network states because doing so results in greater similarity between the firing patterns of oscillators within a temporally-regular group.

**Fig 2:**
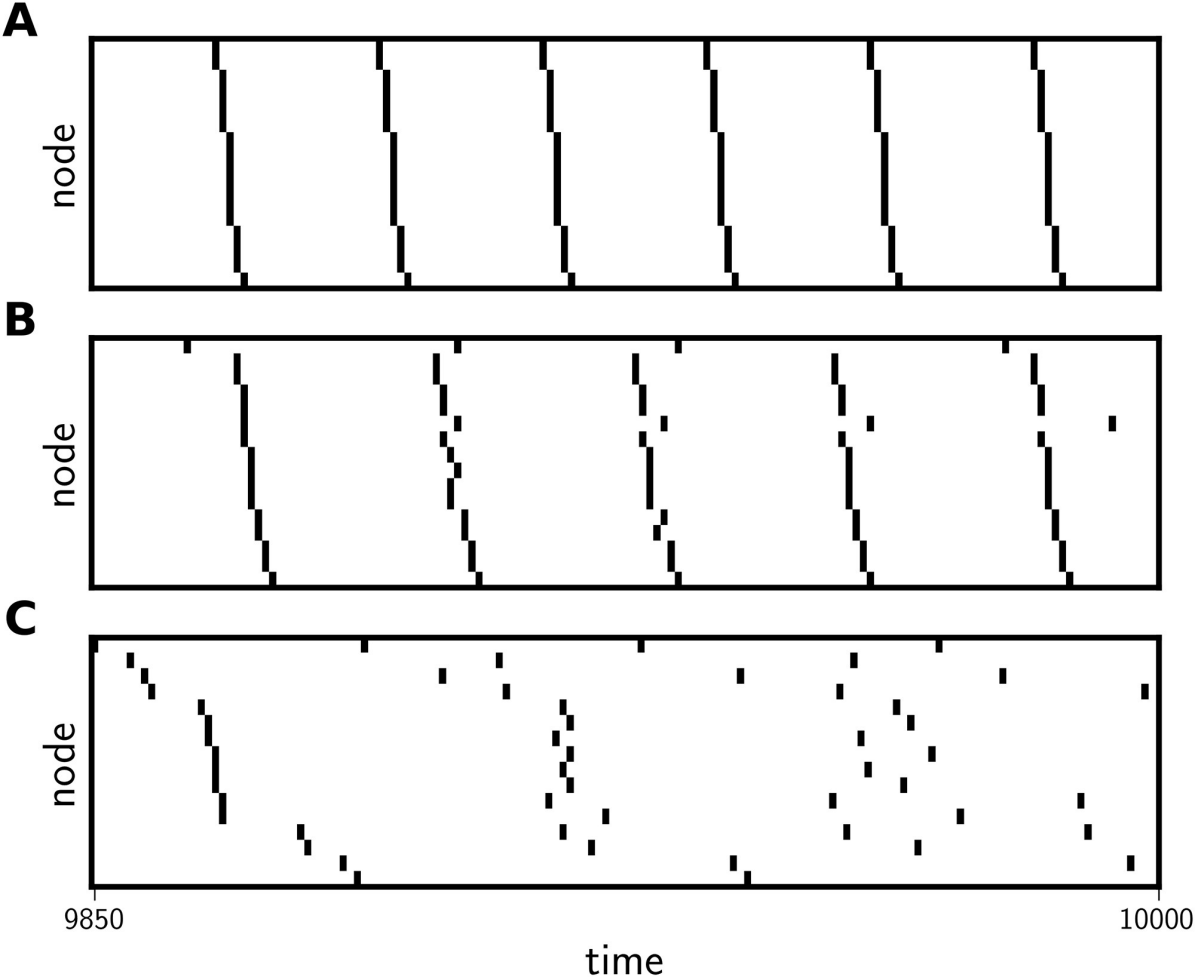
Spike arrays from temporally-regular, chimeric, and temporally-irregular network states. Spike arrays showing the last 150 time-steps of three different network states categorized by ISI statistics as temporally-regular (A), chimeric (B), and temporally-irregular (C). Nodes are sorted by increasing time to the first spike. Compare the perfect timing (all spikes occur with the exact same ISI) in **A** to the random spiking in **C**.

Fig 2 illustrates the patterns of spiking associated with these three broad classes of activity. We find that temporally-regular behavior manifests in the form of oscillators that fire at the same uniform intervals with different temporal offsets (Fig 2A), and not in the form of all oscillators firing in unison. In fact, we have yet to observe this latter pattern in our simulations. Therefore, we support the idea that coordinated activity in pulse-coupled oscillators is better measured by communities of oscillators that share the same ISI statistics than by the existence or absence of uniform locking to a single phase.

### Frequency of network classes

**Fig 3:**
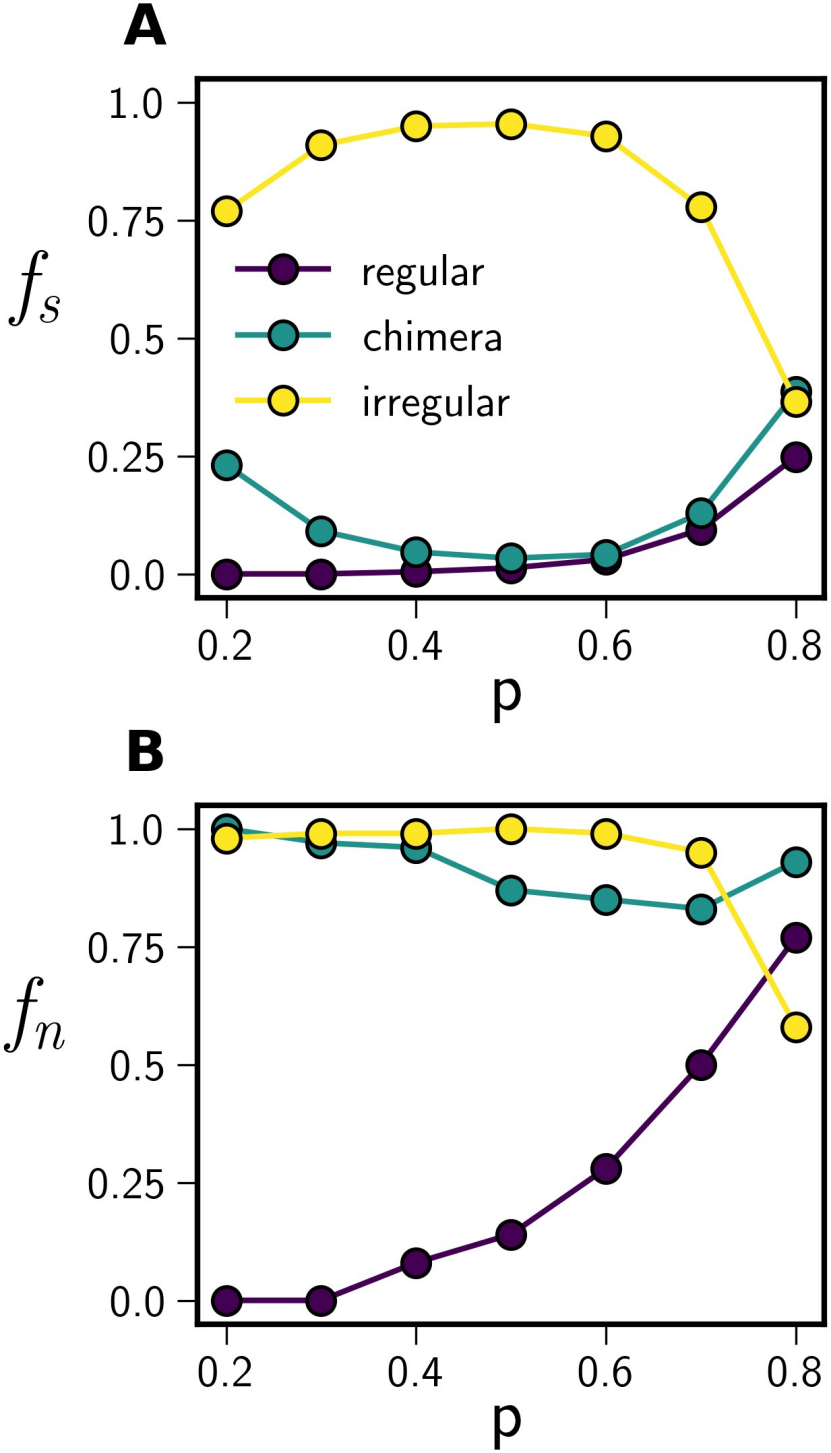
Effect of edge density on state frequencies. One-hundred networks with 500 different initial conditions each were generated with edge densities (*p*) between 0.2 and 0.8. The frequencies *f*_*s*_ in **A** represent the fraction of all 50,000 network states generated at each *p* value that were classified as temporally-regular, chimeric, or temporally-irregular by ISI statistics. The frequencies *f*_*n*_ in **B** are the fraction of 100 networks that produced at least one temporally-regular, chimera, or temporally-irregular state. Temporally-regular and temporally-irregular are abbreviated “regular” and “irregular,” respectively.

We next examine the fraction of networks and initial conditions that exhibit a given class of dynamics as we vary the mean network connectivity *p*. With sparse connectivity, the network topology begins to approximate a chain, which we found to exhibit temporally-regular dynamics in 97% of the 10^5^ network states we tested and form a chimera state in the other 3%. With dense connectivity, the topology approaches a fully connected network, which we found to exhibit temporally-regular dynamics in all of the 10^5^ network states we tested. For intermediate values of *p* in the range [0.2, 0.8], we find that the fraction of chimera states is a convex function of *p* with a minimum at *p* = 0.5 (Fig 3A).

We also find that the frequency of temporally-regular network states increases with network edge density. This result agrees with previous work that found a negative correlation between edge density and time-to-synchronization [23, 24], except that our definition of synchrony encompasses a broader range of network dynamics beyond all oscillators firing simultaneously. Conversely, we find that the frequency of temporally-irregular network states decreases with increasing edges density.

### Network size scaling of class frequencies

We repeated these same simulations, now varying both the network size and the connection density. We varied *N* between 16 and 416 and *p* between 0.2 and 0.8. For each value of *N* and *p*, we generated 100 binomial random networks. We again simulated network dynamics over 10,000 time-steps and categorized the final 2,000 time-steps of the simulation using ISI analysis (as described previously).

**Fig 4:**
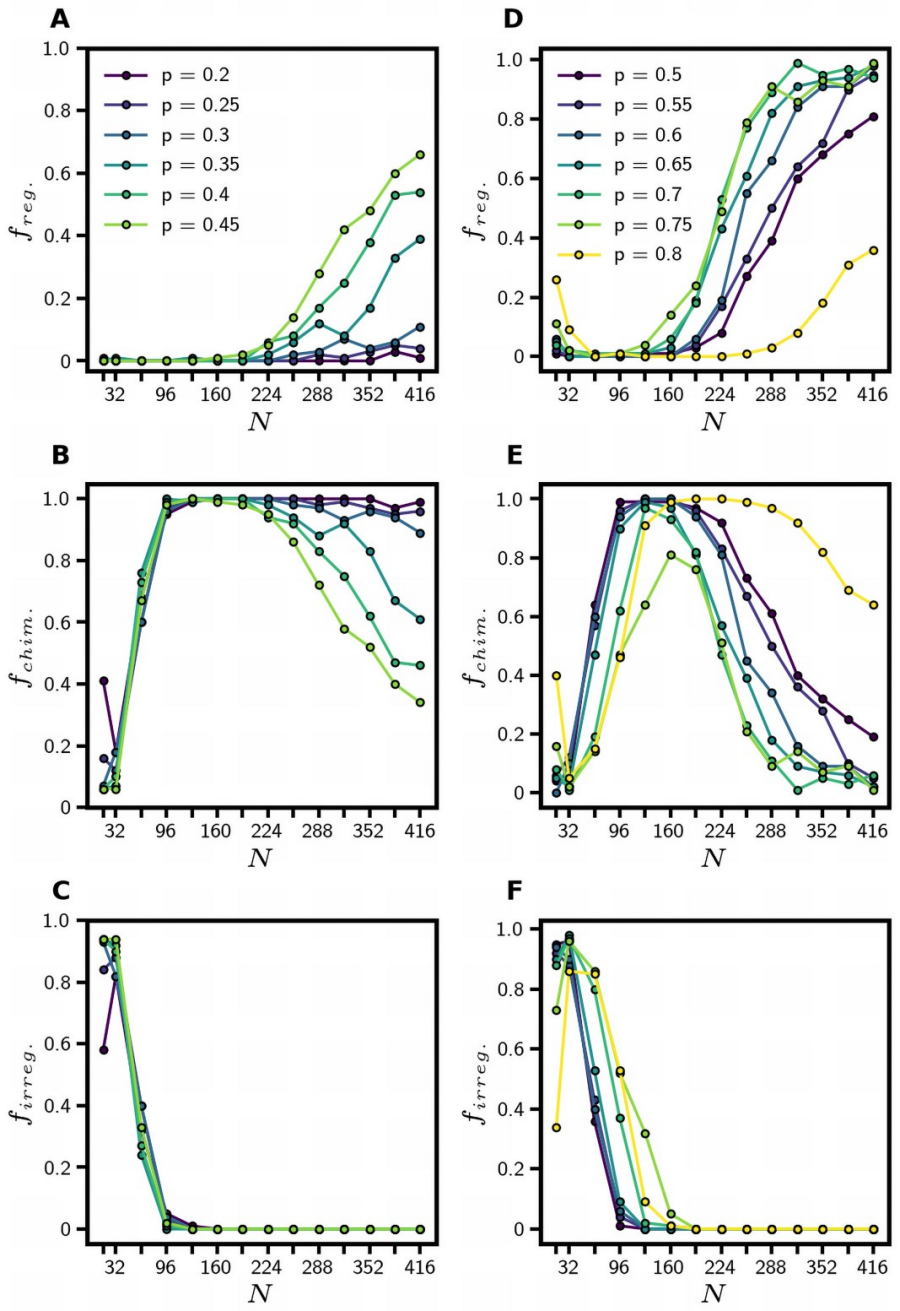
Effect of network size on state frequencies. Panels **A-C** show the frequency of temporally-regular, chimera, and temporally-irregular states with network size *N* and *p* = 0.2, 0.25, …, 0.45. Each point represents a fraction of 100 networks whose dynamics were generated from random initial conditions; this means that the frequency of each state for a single value of *N* and *p* should add to one. Panels **D-F** show the frequency of the three dynamical classes for the same range of *N* and *p* = 0.5, 0.55, …, 0.8.

Fig 4 shows how the frequency of each class of dynamics depends on *N* and *p*. Two features of this data deserve mention. First, for any given *N*, temporally-regular network states are found more frequently at higher values of *p*. To note, *p* = 0.8 is an exception to this trend; for large N, we find that the frequency of chimera states peaks near *p* = 0.8 (detracting from the frequency of temporally-regular states). Fig 3 also shows a monotonic increase in temporally-regular states with increasing *p* for *N* = 16. Second, as *N* increases, the frequency of temporally-regular network states increases for all values of *p*. This trend is mirrored by a decrease in temporally-irregular dynamics with increasing *N* for all values of *p*. While the increase in the frequency of temporally-regular dynamics is preserved for all network sizes, more densely connected networks generally show this increase at a faster rate.

### Degree heterogeneity and network class

The dependence of dynamics on network size can also be explained by degree heterogeneity in the network. Binomial random graphs have a binomial degree distribution by construction, so the expected mean degree in these networks is *p*(*N* − 1), and the expected variance is *p*(*N* − 1)(1 − *p*). Hence, the width of the degree distribution *relative to the mean* scales by $1/\sqrt{N}$, meaning that the degree distribution becomes relatively narrower as *N* increases. We will now show that larger variance in the degree distribution favors temporally-irregular dynamics.

Unfortunately, it is not possible to separately manipulate the mean and variance of the degree distribution of Erdös-Rényi networks. To test whether the shape of the degree distribution—specifically the width—has an independent effect on the class of dynamics, we need to generate networks in which the expected mean degree is fixed but the expected degree variance can be systematically adjusted.

In order to do this, we use the network generation method described by Chung and Lu [28] as modified by Olhede and Wolfe [29]. Briefly, for an *N*-node graph, we draw a random sample *p*_1_, …, *p*_*N*_ from some probability distribution *F*(*p*) with support in the unit interval. The probability *p*_*ij*_ that the edge between nodes *i* and *j* will exist is then equal to *p*_*ij*_ = *p*_*i*_ *p*_*j*_. A random graph generated this way will have a degree distribution that is Poisson Binomial (a Poission mixture of binomial distributions with different success rates *p*_*i*_), and the expected network degree and degree variance will be equal to

$$\left\langle k\right\rangle =\left(N-1\right)\mu^{2}$$ (2)

$$\sigma_{k}^{2}={\left(N-1\right)}^{2}\mu^{2}\{\sigma^{2}+\frac{1-(\mu^{2}+\sigma^{2})}{\left(N-1\right)}\},$$ (3)

where *μ* and *σ*^2^ are respectively the mean and variance of the distribution *F*(*p*) [29]. As an example, if $p_{i}=\sqrt{p}$ for all *i*, this method will generate standard binomial random graphs.

We chose a beta distribution for *F*, which has probability density function

$$\begin{array}{c} p\left(x|\alpha ,\beta \right)=\frac{\Gamma \left(\alpha +\beta \right)x^{\alpha -1}{\left(1-x\right)}^{\beta -1}}{\Gamma (\alpha )\Gamma (\beta )}, \end{array}$$ (4)

where Γ(*x*) is the gamma function. The mean and the variance of the beta distribution are

$$\mu =\frac{\alpha }{\alpha +\beta }$$ (5)

$$\sigma^{2}=\frac{\alpha \beta }{{\left(\alpha +\beta \right)}^{2}\left(\alpha +\beta +1\right)}.$$ (6)

As *α* and *β* become large, the beta distribution becomes more narrow. We fix the expected mean degree by setting the mean of the beta distribution to $\sqrt{p}$. This generates an expected mean connectivity of (*N* − 1)*p*, the same as in a binomial random graph with edge probability *p*. When we do this, the variance of the beta distribution will be

$$\begin{array}{c} \sigma^{2}=\frac{p(1-\sqrt{p})}{\alpha +\sqrt{p}}, \end{array}$$ (7)

which demonstrates clearly that the degree distribution of the resulting graph will widen as alpha is decreased (see Eq 3). We will refer to graphs generated via this method as “beta networks” in what follows.

**Fig 5:**
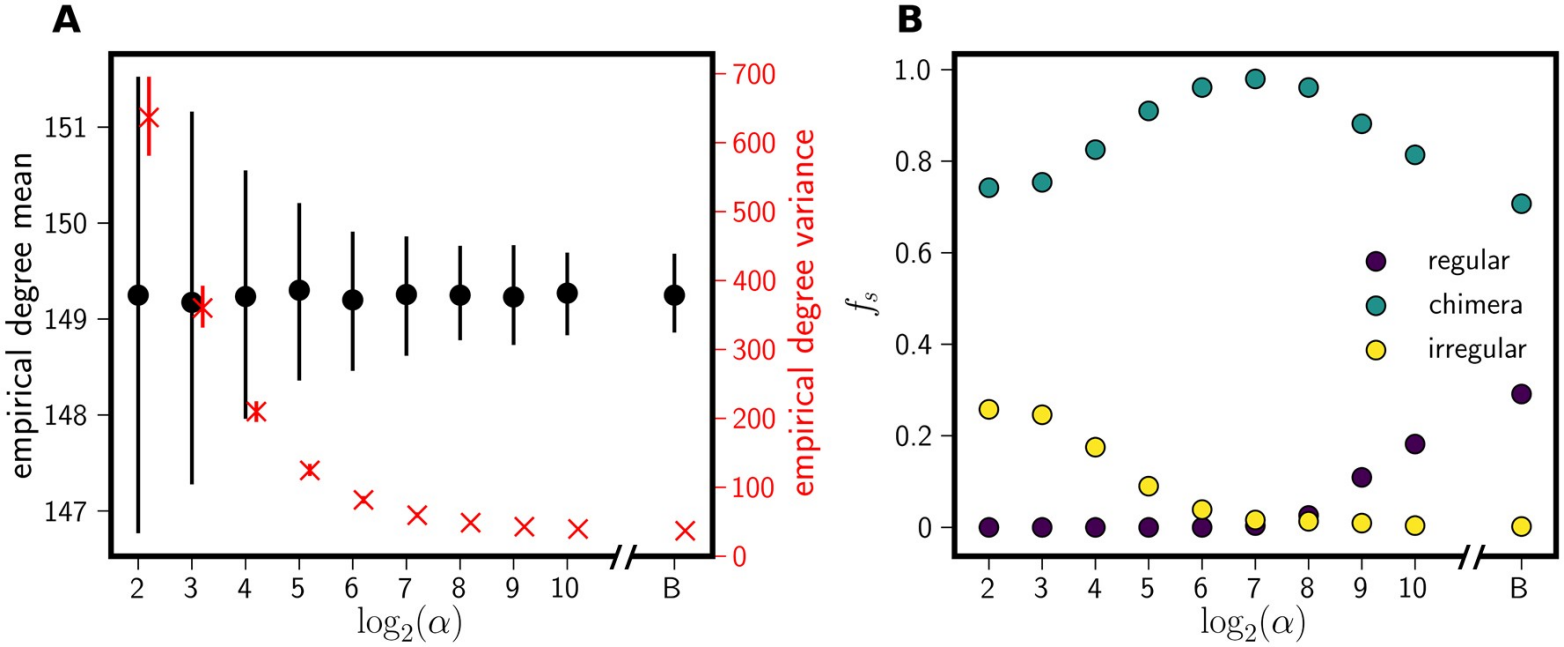
Effect of degree heterogeneity on state frequencies. Panel **A** shows the degree statistics (mean and variance) for 1000 beta networks of size *N* = 200 for each *α* value shown. The mean connectivities were fixed at a value corresponding to *p* = 0.75 in the binomial random graph model. The axis label ‘B’ indicates a binomial random graph with *N* = 200 and *p* = 0.75; otherwise, the tick label indicates the value of *α*. The empirical mean degree is plotted in black (left axis) and the empirical degree variance in red (right axis). In each case, points mark the median ensemble value, and the vertical bars range from the first to third quartiles. The empirical degree variance was shifted slightly to the right to minimize overlap of the data. Panel **B** shows the frequency of each network state generated from the 1,000 beta networks for each *α* value, starting with random initial conditions. As *α* increases, the empirical degree variance decreases resulting in fewer temporally-irregular and more temporally-regular network states.

Fig 5 shows results for ensembles of 1,000 beta networks at varying values of *α*. All networks are of size *N* = 200 and were generated with *p* = 0.75. For comparison, we also include the binomial random graph with *N* = 200 and *p* = 0.75. Fig 5A shows that the empirical mean degree changes by less than one percent as *α* increases, while the empirical degree variance decreases exponentially. Fig 5B shows the frequencies of the three classes of dynamics for each value of *α*. We clearly see a decline in the frequency of temporally-irregular behavior as the expected degree distribution narrows; this is occurring while the mean connectivity changes by less than a percent. We thus conclude that heterogeneity in network degree tends to favor temporally-irregular dynamics.

We also find that the frequency of temporally-regular dynamics increases as the degree distribution narrows, and the frequency of chimeric dynamics reaches a maximum at an amount of variance in the degree distribution slightly larger than the binomial variance. The frequency of chimeric dynamics is already high for *G*(200, 0.75) networks as shown in Fig 4, and it can be further increased by slightly raising the variance of the degree distribution. These findings demonstrate that dynamical patterns are strongly influenced by the expected mean and variance of the network degree distribution.

### Normalized compression distance analysis

While ISI statistics are useful for characterizing spiking dynamics, we require that they be precisely equivalent to eight decimal places in order to identify synchronized. We thus sought a more robust measure that could be used to analyze experimental data that might be corrupted by noise. We introduce the use of NCDs [30] as an alternative method for analyzing dynamics.

Normalized compression distance is a universal similarity metric intended to approximate the normalized information distance, which relies on noncomputable Kolmogorov complexities. With the following formula, we can calculate the distance between two sequences *x* and *y*:

$$\begin{array}{c} \text{NCD}\left(x,y\right)=\frac{C(xy)-\text{min}\{C(x),C(y)\}}{\text{max}\{C(x),C(y)\}}, \end{array}$$ (8)

where *xy* denotes the concatenation of the two sequences, and the function *C*(⋅) returns the length in bytes of the compressed sequence argument. Hence, NCD(*x*, *y*) measures the difference between the compressed lengths of the concatenated sequences and the shortest compressed individual sequence. If *x* and *y* are completely unrelated, then *C*(*xy*) will be much larger than *C*(*x*) or *C*(*y*), and NCD(*x*, *y*) will be close to one. If *x* and *y* are largely redundant, then concatenating the two adds no extra information, and the compressed length of *xy* will be similar to min{*C*(*x*), *C*(*y*)}, resulting in an NCD(*x*, *y*) value close to zero.

NCDs have been used to assess the similarity of musical pieces [31], text documents [32], and SMILES representation of molecules [33]. With its wide range of uses, the efficacy of the NCD formula depends critically on the compatibility of the data with the compression algorithm used to compute *C*(⋅). For any given application, the compressor must satisfy the following properties [34]:

- Idempotency: *C*(*xx*) = *C*(*x*)
- Symmetry: *C*(*xy*) = *C*(*yx*)
- Monotonicity: *C*(*xy*) ≥ *C*(*x*)
- Distributivity: *C*(*xy*) + *C*(*z*) ≤ *C*(*xz*) + *C*(*yz*)

In order to identify the most accurate method for calculating the NCD values of spike trains (binary sequences), we measured all of these properties in multiple compressors. We found no violations of distributivity or monotonicity using any of the compressors. We consider idempotency to be the most relevant property for capturing the structure of our data because it directly indicates the ability of a compressor to detect shared information, a hallmark of synchronous activity.

We tested two different implementations of prediction by partial matching that use arithmetic encoding [35]. We also tested the Python gzip and bz2 compressors. Python gzip uses a variant of the Deflate algorithm that involves combining the calculation of Lempel-Ziv complexity [36] with Huffman encoding [37]. Python bz2 combines a Burrows-Wheeler transform with Huffman encoding. Finally, we tested Google’s Snappy compressor which is optimized for performance and does not use an entropy encoder at all.

**Fig 6:**
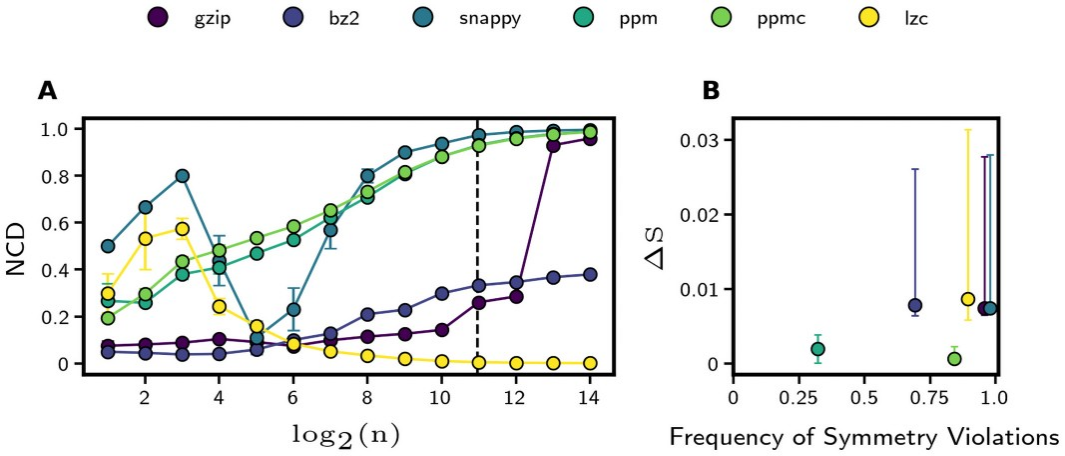
Compressor idempotency and symmetry. Five different compression algorithms were tested for idempotency and symmetry in the normalized compression distance metric: the widely used “gzip” and “bz2,” two different prediction by partial matching implementations “ppm” and “ppmc,” and Google’s “Snappy.” The Lempel-Ziv complexity was also used as a proxy for compressed length in NCD calculation. Panel **A** shows the average NCD between ten binomial random sequences (*p* = 0.5) and themselves for different sequence lengths (ideally, the NCD should be close to zero). Error bars indicate the standard error of the mean. The dashed vertical line indicates the length of the spike trains analyzed in the rest of this study. Panel **B** shows size of symmetry violation (see Eq 9) against the frequency of a violation for 1,000 different pairs of binomial random sequences (*p* = 0.5). Error bars indicate 95% confidence intervals.

Most of the compressors demonstrated poor idempotency, as indicated by high NCD values between a sequence and itself, especially for long sequences (Fig 6A). The gzip and bz2 Python modules outperformed both the prediction by partial matching algorithms and Google’s snappy library with Python bindings; however, idempotency of the NCDs measured by gzip begins to decline for sequences longer than 2^15^ bits (2^9^ sequence elements). This may have to do with the sequence length exceeding gzip’s window size.

To preserve idempotency and eliminate the need for sequences to be shorter than 2^15^ bits, we introduced the use of Lempel-Ziv complexity (LZC) to calculate the NCDs between sequences [36]. This substitution forms a distance metric similar to a formula previously used for phylogenic tree construction with DNA nucleotide sequences [38]. LZC is a well-known approximation of the Kolmogorov Complexity, and it has been successfully used to estimate the entropy of binned spike trains [39]. Moreover, LZC is the basis of another compression algorithm (LZW) [40, 41], so there is a clear link between LZC and the compressed size of a sequence. Importantly, when we calculated NCD using *LZC*(⋅) in place of *C*(⋅), the NCD between a sequence and itself converges to zero with increasing sequence length, which signifies that the idempotency property is satisfied (Fig 6A).

We used the following expression to calculate the symmetry Δ*S* of compressing two sequences *xy* and *yx* given *x* and *y* as a fraction of the maximum compressed length:

$$\begin{array}{c} \Delta S\left(x,y\right)=\frac{\left|C(xy)-C(yx)\right|}{\text{max}\{C(xy),C(yx)\}}. \end{array}$$ (9)

This normalization is consistent with that of the NCD metric. Values of Δ*S* greater than zero indicate symmetry violations.

Most of the compressors frequently exhibited symmetry violations, but the average extent of those violations Δ*S* was not concerning (Fig 6B). Using LZC, symmetry was violated in 90% of sequence comparisons (*xy* and *yx* for some *x* and *y*) but $\bar{\Delta S}<0.01$. Though we find that prediction by partial matching compressors have greater symmetry while processing our data, their lack of idempotency makes them inadequate for the rest of our study. Prediction by partial matching compressors are also typically more computationally expensive than other compressors, and much more so than simple LZC calculation.

Finally, we compared NCD values between binomial random and periodic sequences as the sequence length increased. As expected, when using LZC to calculate NCD values, the NCD values of binomial random sequences converged to unity, and the NCD values of periodic sequences stabilized at a smaller value independent of sequence length. LZC was the only compressor that exhibited this behavior (not shown).

**Fig 7:**
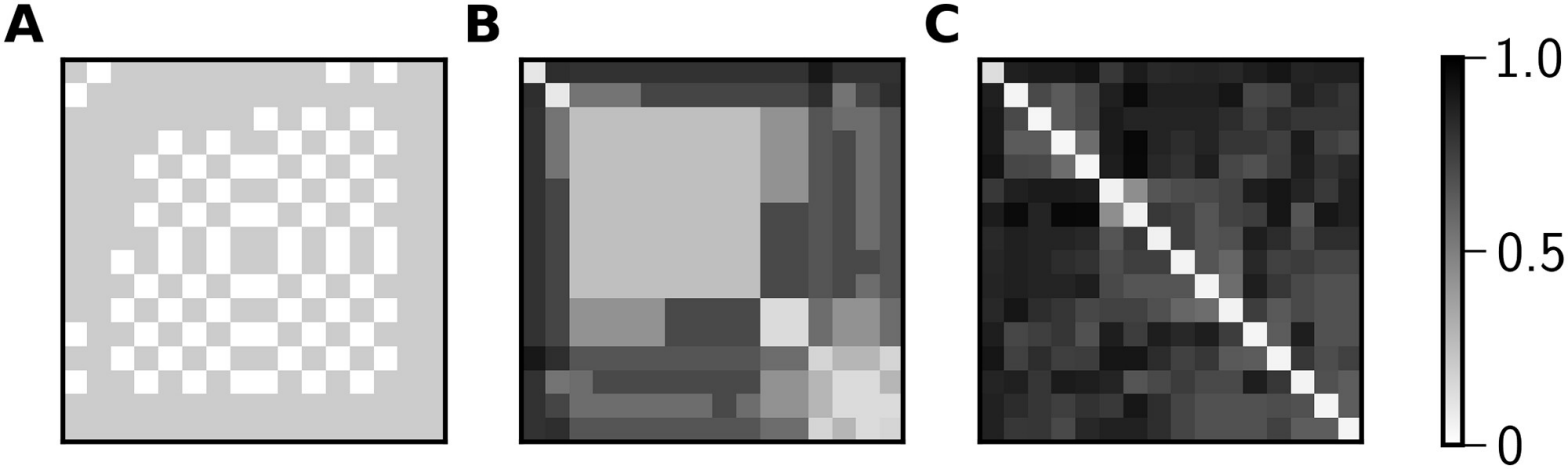
NCD matrices of temporally-regular, chimeric, and temporally-irregular network states. Calculation of the pairwise NCDs for the 16 nodes of the spike arrays shown in Fig 2 yields matrices that are indicative of three different network states. Both by ISI statistics and NCD, **A** is a temporally-regular state, **B** is a chimera state, and **C** is a temporally-irregular state.

We used LZC to compute pairwise NCD values between all pairs of spike trains produced by nodes in a given network; Fig 7 shows examples of typical NCD matrices for the three states shown in Fig 2. We investigated many properties of these pairwise NCD matrices—mean, variance, degree of bimodality, and singular value spectrum—with the goal of identifying properties that distinguish matrices associated with the three broad classes of network behavior that we observed. However, we conclude that the best way to use NCD values to delineate between the three broad classes of dynamics is as follows:

1. **Temporally-irregular**: all pairwise NCD values are greater than the threshold of 0.3
2. **Temporally-regular**: all pairwise NCD values are less than or equal to the threshold of 0.3
3. **Chimeric**: at least one pairwise NCD value is above 0.3, and at least one pairwise NCD value is below 0.3

The threshold value of 0.3 was chosen such that the NCD classifications had maximal overlap with ISI classification. 92% of 35,000 network states sampled from Fig 3 were classified the same (Table 1). Raising the threshold value for NCD classification increases the percentage of network states classified as temporally-regular by both methods, but decreases the number of network states classified as temporally-irregular by both methods. The fact that we find consistent classification results using NCD and ISI analyses indicates that both methods can be used to identify dynamical patterns in our simulated spike array data. Much like ISI analysis, NCD matrices illustrate which nodes are synchronized (low pairwise NCDs) and which are not (high pairwise NCDs), as shown in Fig 7.

**Table 1:** Network state classification by ISI statistics and NCD values.

		**NCD category**		
		regular	chimera	irregular
**ISI category**	regular	0.03	0.01	0
	chimera	0.00	0.13	0.01
	irregular	0	0.06	0.76

Comparison of categorization with ISI statistics and NCD using 35,000 network states consisting of a random sample of 50 networks and 100 corresponding initializations for each *p* in Fig 3. The fraction of network states with the corresponding ISI and NCD categorizations are reported in each cell. Network states classified the same by both methods are shown on the diagonal while network states categorized differently are on the off-diagonal. Numbers on the diagonal should be compared to values in the same row or column, as the sample is highly unbalanced—the vast majority of states in this sample are temporally-irregular.

**Fig 8:**
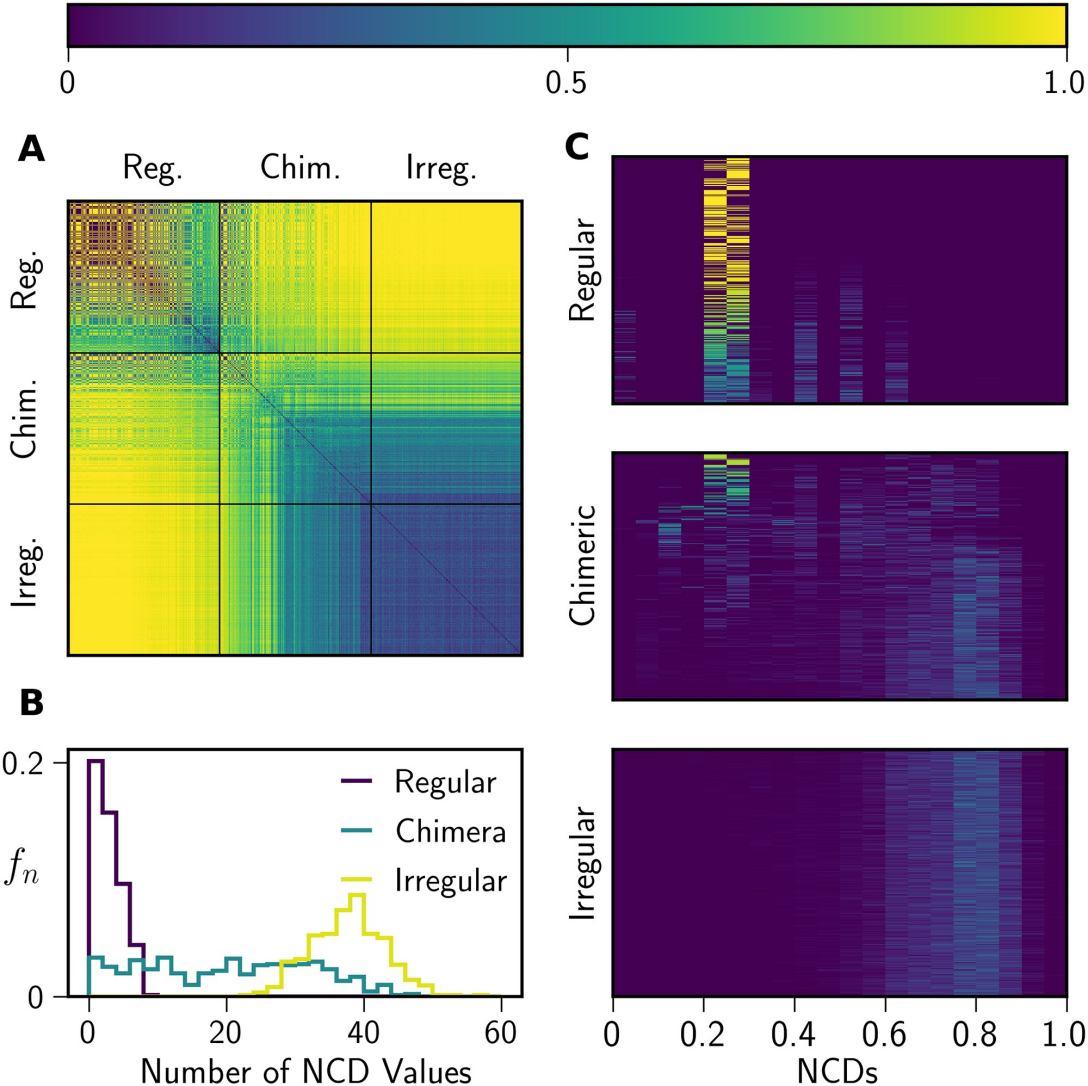
NCD distributions of 1,260 different network states. Panel **A** shows the pairwise Jensen-Shannon distances (JSDs) between the NCD distributions of 1,260 different network states. Thick black lines separate the three different classes of network behavior as determined by ISI statistics. The JSDs within each block are sorted by increasing NCD distribution entropy from left to right. For the chimera states, 30 network states with each number of synchronized nodes (2 to 15) are shown. Panel **B** shows the distribution of the number of NCD values for each network state in **A**, and panel **C** shows the distributions of NCD values that are compared pairwise in the Jensen-Shannon distance matrix.

One of our main findings is that any attempt to separate these states with perfect accuracy is *a priori* unachievable, because chimera states are not characterized by a single set of unique dynamics. Instead, they exhibit dynamics that smoothly transition between temporally-regular and temporally-irregular dynamics (Fig 8).

To demonstrate this transition, we computed Jensen-Shannon distances between the NCD distributions of 1,260 network states (420 states from each class, as determined by ISI statistics). Within the chimeric class, we ensured that there was a total of 30 states that exhibited synchronized dynamics among *n* of *N* nodes, with *n* = 2, …, 15. Fig 8A shows the JSDs between all pairs of network states. The large JSDs between temporally-regular and temporally-irregular states indicate the clear difference between their underlying dynamical patterns, as captured by their NCD distributions. We find that chimera states with more synchronized nodes have pairwise NCD patterns that are more similar to those of temporally-regular states, while chimera states with fewer synchronized nodes exhibit pairwise NCD patterns that are more similar to temporally-irregular states. These trends illustrate how chimera states exhibit a range of different dynamical patterns that interpolate between temporally-regular and temporally-irregular dynamics.

Finally, high JSDs among temporally-regular states indicate that these states do not manifest as a single, monolithic synchronous network state (i.e., all nodes firing at the same time-step), but rather exhibit differences in their distributions of NCD values. The NCD distributions of temporally-regular states usually have a small number of unique values, often only one NCD value. These NCD values differ because temporally-regular network states have different mean ISIs, although there is not a one-to-one relationship between a set of mean ISI values and a set of pairwise NCD values. Differences in narrow distributions of NCD values result in large JSDs between different temporally-regular states, even though the underlying dynamics of temporally-regular states are more similar to each other than they are to states in the two other classes.

### Behavior in other random graphs

**Fig 9:**
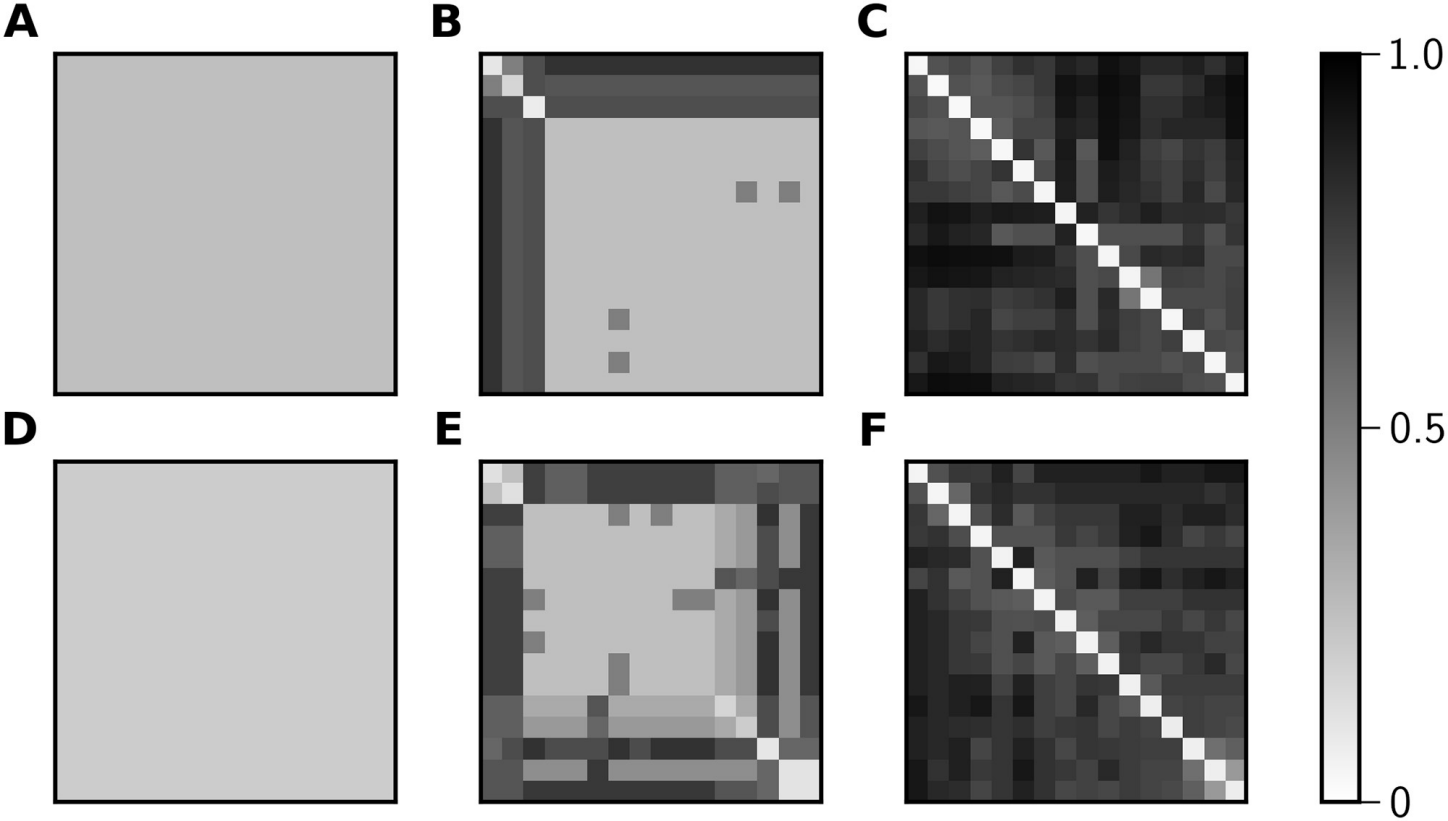
Temporally-regular, chimeric, and temporally-irregular states in other random graph ensembles. Panels **A-C** show sample NCD matrices for Barabási-Albert pulse-coupled oscillator networks with 16 nodes. The mean degree in these networks was drawn randomly from a uniform distribution with range [3,15). According to both ISI and NCD-based categorization, **A** is a temporally-regular state, **B** is a chimera state, and **C** is a temporally-irregular state. Panels **D-F** show sample NCD matrices for states of 16-node Newman-Watts-Strogatz networks. Each node is connected to its two nearest neighbors and additional edges were added with probability drawn randomly from a uniform distribution with range [0.2,0.8). Similar to the first row, (**D**) is a temporally-regular state, (**E**) is a chimera state, and (**F**) is a temporally-irregular state.

In order to verify that the coexistence of these three dynamical states is not specific to Erdös-Rényi graphs, we characterized the dynamical states in other well-known random graphs, Barabási-Albert [42] and Newman-Watts-Strogatz [43, 44]. We find examples of all three network states in both network topologies (Fig 9), which can be classified similarly by ISI and NCD (Tables 2 and 3). This suggests that NCDs could be a useful strategy for analyzing real-world networks, which often exhibit the characteristics of multiple idealized network models (i.e., the power law degree distribution of Barabási-Albert networks and the high clustering coefficient of Newman-Watts-Strogatz networks). Moreover, observing these states in other random graph ensembles suggests that they can coexist in phase space even in large graphs. While for binomial random graphs the degree fluctuations relative to the mean decay like $1/\sqrt{N}$ as the size of the graph increases, this is not true in graphs with power law degree distributions like the Barabási-Albert network.

**Table 2:** Network state classification by ISI statistics and NCD values in Barabási-Albert network states.

		**NCD category**		
		regular	chimera	irregular
**ISI category**	regular	0	0	0
	chimera	0	0.15	0.00
	irregular	0	0.01	0.84

Comparison of categorization with ISI statistics and NCD using 35,000 Barabási-Albert network states. 100 networks were generated for each *m* = 2, 3, …, 8, and 50 states were produced from each network with different random initial conditions. The overall agreement calculated as the sum of the diagonal over the total sum is 98.9%.

**Table 3:** Network state classification by ISI statistics and NCD values in Newman-Watts-Strogatz network states.

		**NCD category**		
		regular	chimera	irregular
**ISI category**	regular	0.23	0.26	0
	chimera	0	0.19	0.01
	irregular	0	0.07	0.25

Comparison of categorization with ISI statistics and NCD using 35,000 Newman-Watts-Strogatz network states. 100 networks were generated for each combination of *p* and *k* that resulted in an approximate connection density of 0.2, 0.3, …, 0.8. These combinations were (*p*, *k*) = (0.5, 2), (0.1, 4), (0.5, 4), …, (0.5, 8). 50 states were then produced from each network with different random initial conditions. The overall agreement calculated as the sum of the diagonal over the total sum is 66.9%.

## Discussion

This study introduces two methods for identifying dynamical patterns in pulse-coupled oscillator networks. We find a form of structured activity (“temporally-regular”) in which nodes fire on predictable intervals, but not necessarily in phase with one another (Fig 2A). These regular network states can also be identified by their low pairwise NCD values among nodes and small number of unique pairwise NCDs (Figs 7A and 8B). We also identified a second form of structured activity that is consistent with previous definitions of a chimera state. In these network states, at least one group of nodes fires coherently while the rest of the nodes in the network fire without any recognizable pattern (Fig 2B). These network states typically have both high (> 0.3) and low (< 0.3) NCDs between nodes (Fig 7B), and the distribution of NCD values is correlated with the number of nodes that fire on the same interval (Fig 8C). Using ISI statistics, we have found cases where two or more group of nodes are synchronized but exhibit different intra-group dynamics; such states have been previously identified as “multichimera states” [13]. We also observe network states without any coordinated activity that can be identified by either ISI or NCD analysis, and we refer to these states as temporally-irregular.

Previous work on pulse-coupled oscillators found long, disordered transients with positive maximum Lyapunov exponents estimated analytically [23]. This suggests that our temporally-irregular states are likely chaotic. However, more remains to be done on this point.

While we identify three broad dynamical patterns with both ISIs and NCDs, both of these classification methods indicate that the patterns form a continuum of dynamical behavior rather than classes that can be perfectly separated. This is consistent with previous work that used measures such as the order parameter [11, 13, 16] or curvature [20] to quantify the degree of synchronization on a continuous scale and then partition this continuum into discrete network states.

There are benefits and drawbacks to using either ISIs or NCDs to characterize network state dynamics. We proposed NCDs as a method of analyzing spike arrays that may exhibit synchronous activity but that does not manifest as sets of nodes with precisely identical ISI statistics (potentially due to noise). However, calculating NCDs is much more computationally expensive. Parallelizing network state analysis using either method greatly reduces runtime, but the runtime of the NCD calculation is very sensitive to the compression algorithm and its specific implementation. We have shown that the two methods agree on the majority of network state classifications, but neither can be considered a “ground truth” synchronization metric, and thus either may be useful depending on the data one wants to analyze.

We found that edge density and variance of expected degree distribution impacted the tendency of Erdös-Rényi graphs to exhibit different classes of dynamical behavior (Figs 3–5). Overall, greater variance in degree distribution and intermediate edge densities resulted in primarily temporally-irregular dynamics while narrower degree distributions and high edge densities resulted in primarily temporally-regular dynamics. There appears to be a range of *N* for our networks that produces a higher frequency of chimera states for all *p*, and this range separates the high frequency of temporally-irregular dynamics at low *N* and the high frequency of temporally-regular dynamics at high *N*. Consequently, it can be inferred that moderate amounts of variance in the expected degree distribution likely favor chimera states. It remains unknown how edge density and other graph statistics impact these classes of dynamics in other network ensembles. Barabási-Albert networks are known to be sparsely connected and have a scale-free degree distribution, so we hypothesize that chimeric and temporally-irregular dynamics will more frequently arise in these networks.

Another study that addresses the relationship between network size and dynamics with a theta neuron oscillator model also found a higher frequency of temporal regularity in larger networks [21]. This study focused on networks of 5,000 phase oscillators and used the Kuramoto order parameter [6] to identify temporally-regular dynamics. Our methods of categorizing networks of pulse oscillators are quite different—they more intuitively identify subgroups of synchronized nodes that make up chimera states, and they characterize discrete, rasterized data—but they also find an increase in temporally-regular dynamics with *N*. Two studies that measure the time-to-synchronization *T* of communities of pulse oscillators have found a dependence of *T* on *N*. One study claims that there is a power law relationship between *T* and *N* [24], and another claims that *T* increases exponentially with *N* [23], but this was found in the range *N* = [10, 25] and with *p* = 0.8. We too find a sharp increase in irregular dynamics within the range *N* = [16, 32], and a sharp decrease following *N* = 32, which agrees with both of these findings if we consider low *T* congruent with temporally-regular dynamics (Fig 4).

In this study, we assumed that delays in signal propagation were infinitesimal. In future work, it would be interesting to determine how finite time delays interact with network topology to shape collective dynamics. One study found that globally connected pulse oscillators with the same phase resetting curve fired in unison for some range of delay values, and that both excitatory and inhibitory networks can achieve a synchronous state where all oscillators fire periodically in unison for a delay in that range [45]. It is not yet fully understood how delays or inhibitory connections (we considered only excitatory) impact the distribution of dynamical classes within this model, especially when considering that there are different ways of identifying dynamical patterns.
